# Patterns of postictal cerebral perfusion in idiopathic generalized epilepsy: a multi-delay multi-parametric arterial spin labelling perfusion MRI study

**DOI:** 10.1038/srep28867

**Published:** 2016-07-04

**Authors:** Guangxiang Chen, Du Lei, Jiechuan Ren, Panli Zuo, Xueling Suo, Danny J. J. Wang, Meiyun Wang, Dong Zhou, Qiyong Gong

**Affiliations:** 1 Huaxi MR Research Center (HMRRC), Department of Radiology, West China Hospital of Sichuan University, Chengdu, Sichuan Province, China; 2 Department of Radiology, Affiliated Hospital of Luzhou Medical College, Luzhou, Sichuan Province, China; 3 Department of Neurology, West China Hospital of Sichuan University, Chengdu, Sichuan Province, China; 4 Siemens Healthcare, MR Collaborations NE Asia, Beijing, China; 5 Department of Neurology, UCLA, Los Angeles, CA, USA; 6 Department of Radiology, Henan Provincial People’s Hospital & the People’s Hospital of Zhengzhou University, Zhengzhou, Henan Province, China

## Abstract

The cerebral haemodynamic status of idiopathic generalized epilepsy (IGE) is a very complicated process. Little attention has been paid to cerebral blood flow (CBF) alterations in IGE detected by arterial spin labelling (ASL) perfusion magnetic resonance imaging (MRI). However, the selection of an optimal delay time is difficult for single-delay ASL. Multi-delay multi-parametric ASL perfusion MRI overcomes the limitations of single-delay ASL. We applied multi-delay multi-parametric ASL perfusion MRI to investigate the patterns of postictal cerebral perfusion in IGE patients with absence seizures. A total of 21 IGE patients with absence seizures and 24 healthy control subjects were enrolled. IGE patients exhibited prolonged arterial transit time (ATT) in the left superior temporal gyrus. The mean CBF of IGE patients was significantly increased in the left middle temporal gyrus, left parahippocampal gyrus and left fusiform gyrus. Prolonged ATT in the left superior temporal gyrus was negatively correlated with the age at onset in IGE patients. This study demonstrated that cortical dysfunction in the temporal lobe and fusiform gyrus may be related to epileptic activity in IGE patients with absence seizures. This information can play an important role in elucidating the pathophysiological mechanism of IGE from a cerebral haemodynamic perspective.

Idiopathic generalized epilepsy (IGE) is characterized by typical absence seizures, generalized tonic-clonic seizures, juvenile myoclonic epilepsy and paroxysmal generalized spike and wave (GSW) discharges on electroencephalography (EEG)^1^. Patients with IGE display all or some of these seizure subtypes. IGE with absence seizure is a special subtype of epilepsy given a paroxysmal loss of consciousness during the sudden onset and sudden end with or without generalized tonic-clonic seizures and juvenile myoclonic epilepsy. Although it is generally accepted that no neuroimaging abnormalities are present in IGE, image processing and quantitative magnetic resonance imaging (MRI) studies suggest that subtle structural and functional abnormalities may exist^2^.

Arterial spin labelling (ASL) is a non-invasive magnetic resonance perfusion approach to the measurement of cerebral blood alterations by magnetically labelling the inflowing water proton spins in the arterial blood proximal to the tissue of interest as a freely diffusible tracer^3^. Due to its complete non-invasiveness and lack of radiation exposure, ASL perfusion MRI has been increasingly applied to investigate the perfusion patterns in both healthy subjects and patients^4^,^5^,^6^. In epilepsy, ASL is mainly applied to characterize the ictal and interictal cerebral blood flow (CBF) alterations for localizing the epileptic focus, especially for these subjects without abnormal findings in structural MRI or other examinations^7^,^8^,^9^,^10^,^11^. Overall, most studies have revealed hyperperfusion during the periictal period and hypoperfusion during the interictal period^7^,^8^,^12^,^13^. Moreover, ASL has a very good concordance with fluorodeoxyglucose positron emission tomography (FDG-PET) and a moderate concordance with video-EEG monitoring^8^,^14^.

However, most ASL techniques in previous studies have acquired images at a single-delay time point. The main limitation of single-delay ASL is that the delay time between labelling in the feeding arteries and arrival of labelled blood in tissue (i.e., arterial transit time or ATT) can have a large effect on the perfusion signals^15^. ASL perfusion MRI can suffer from artefacts and quantification errors when the delay time between labelling and arrival of labelled blood in the tissue is uncertain^16^. Therefore, the selection of a single, optimal delay time is difficult because the ATT varies widely among patients with different vascular and perfusion characteristics^16^,^17^.

The recently developed method of multi-delay multi-parametric ASL perfusion MRI allows the limitations of single-delay time point studies to be overcome. It has several potential advantages over existing single-delay ASL perfusion MRI, including improved accuracy of CBF quantification and imaging of multiple haemodynamic parameters (ATT, CBF and arterial cerebral blood volume or aCBV)^18^. Moreover, by combining single-shot three-dimensional (3D) GRASE (gradient and spin echo), background suppression and pseudo-continuous ASL (pCASL), the temporal stability of multi-delay ASL has been dramatically improved within a clinically reasonable total scan time^15^,^19^. The technique has been employed successfully in cerebrovascular diseases, such as acute ischemic stroke^18^ and moyamoya disease^15^, which demonstrates significant correlations between multi-delay ASL and dynamic susceptibility contrast (DSC) as well as computed tomography (CT) perfusion CBF measurements. In addition, a recent study using this protocol found no significant differences in CBF and significantly prolonged ATT between patients with late-onset epilepsy and control subjects^20^. The authors considered that the prolonged ATT may be attributable to the recruitment of secondary collaterals induced by seizure activity. Nevertheless, further research is needed to confirm this notion.

The cerebral haemodynamic status is a very complicated process before, during, and after the epilepsy status of IGE. Therefore, characterization of the cerebral perfusion patterns in patients with IGE at different periods of epilepsy is necessary to better understand the fundamental underlying mechanisms of IGE. To date, the cerebral perfusion patterns of IGE patients have largely been derived from these studies using PET^21^ or single photon emission computed tomography (SPECT)^22^,^23^,^24^,^25^, and little attention has been paid to the cerebral blood alterations detected by ASL perfusion MRI in IGE patients. Moreover, published studies of cerebral blood alteration in patients with IGE have produced inconsistent results. An H_2_^15^O PET study revealed a mean global increase of 14.9% in CBF in association with typical absences. In addition to the global increase, a focal increase in thalamic blood flow of 3.9 to 7.8% following hyperventilation-induced typical absences with GSW discharges was noted^21^. Measurements of CBF using transcranial Doppler ultrasonography in absence seizures^26^ and SPECT in IGE^22^ and childhood absence epilepsy^23^,^24^ revealed ictal increase and postictal decrease. Conversely, a SPECT study reported that CBF is diffusely reduced throughout the brain during the occurrence of typical absence seizure, whereas CBF increases during the postictal phase^27^. Another SPECT study in patients with juvenile myoclonic epilepsy during the interictal period demonstrated a significant regional CBF reduction in bilateral thalami, cerebelli and the brainstem, whereas regional CBF increased in the left superior frontal gyrus^25^.

The above studies indicate complex changes of CBF during different phases of IGE, and the perfusion pattern of CBF does not always involve an ictal increase and interictal decrease. Therefore, the purpose of the present study was to conduct an exploratory study for characterizing the patterns of postictal cerebral perfusion in IGE patients with absence seizures using multi-delay multi-parametric ASL perfusion MRI to: (1) investigate whether there are significant differences in the parameters of ATT and CBF using voxel-based whole brain analysis between these patients and healthy control subjects, and (2) evaluate the relationships between these identified neuroimaging findings and illness duration, as well as the age at onset.

## Results

### Characteristics of the participants

All 21 IGE patients with absence seizures and 24 healthy control subjects underwent ASL perfusion MRI at 0.5 to 30 days (mean ± standard deviation (SD): 7.6 ± 5.9 days) since the last epilepsy episode had occurred. The characteristics of the participants are presented in Table 1. The seizure types of the patients included absence seizures in 4 patients, absence seizures with juvenile myoclonic epilepsy in 3, absence seizures with generalized tonic-clonic seizures in 8 and absence seizures with generalized tonic-clonic seizures and juvenile myoclonic epilepsy in 6. All of the patients had GSW discharges on video-EEG.

### Regional cerebral perfusion difference in ATT and CBF

**Figure 1:**
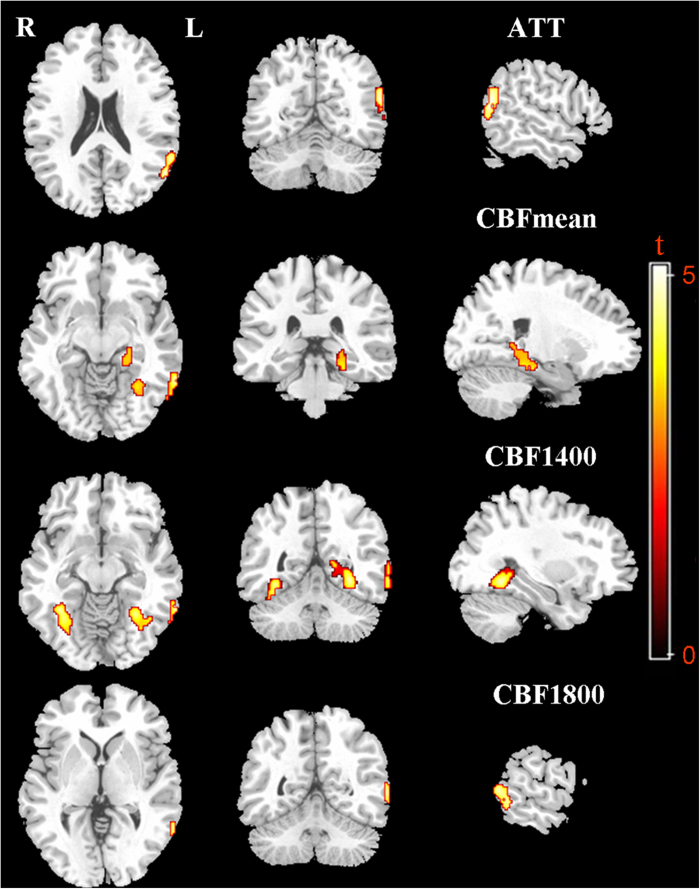
Axial, coronal, and sagittal views of the brain regions with the alterations of cerebral perfusion in IGE patients with absence seizures (FDR corrected *p* < 0.05). The ATT was prolonged in the left superior temporal gyrus. The mean CBF was increased in the left middle temporal gyrus, left parahippocampal gyrus and left fusiform gyrus. The CBF at 1,400 ms was increased in the left parahippocampal gyrus, bilateral fusiform gyri and left middle temporal gyrus. The CBF at 1,800 ms was increased in the left middle temporal gyrus. IGE, idiopathic generalized epilepsy; ATT, arterial transit time; CBF, cerebral blood flow; FDR, false discovery rate.

Patients with IGE showed prolonged ATT in the left superior temporal gyrus versus healthy control subjects (Table 2, Fig. 1). The mean CBF of the IGE patients was significantly increased in the left middle temporal gyrus, left parahippocampal gyrus and left fusiform gyrus (Table 2, Fig. 1). Compared with CBF at 1,800, 2,200 and 2,600 ms, the CBF at 1,400 ms was more sensitive for the detection of CBF changes in the patients with IGE. At 4 post-labelling delay (PLD) times, the CBF at 1,400 ms of the IGE patients was significantly increased in the left parahippocampal gyrus, bilateral fusiform gyri and left middle temporal gyrus; the CBF at 1,800 ms of the IGE patients was significantly increased in the left middle temporal gyrus versus healthy control subjects (Table 2, Fig. 1); however, the CBF at 2,200 and 2,600 ms did not exhibit a significant difference between the IGE patients and healthy control subjects.

### Correlation analyses

**Figure 2:**
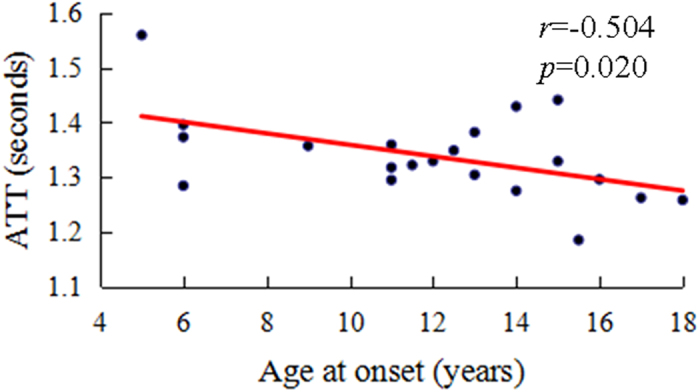
Correlation analysis result between ATT in the left superior temporal gyrus and the age at onset. The prolonged ATT in the left superior temporal gyrus was significantly negatively correlated with the age at onset in IGE patients with absence seizures.

The ATT in the left superior temporal gyrus was negatively correlated with the age at onset in patients with IGE (*r* = −0.504, *p* = 0.020) (Table 3, Fig. 2). No significant correlation was noted between the CBF in these brain areas and illness duration or the age at onset (Table 3).

## Discussion

In the present study, we identified postictal cerebral perfusion alterations in IGE patients with absence seizures relative to healthy control subjects using multi-delay multi-parametric ASL perfusion MRI. The results indicate that the CBF at 1,400 ms among the 4 PLD times was the most sensitive for the detection of CBF changes in IGE patients with absence seizures, with a significantly increased CBF in the left parahippocampal gyrus, bilateral fusiform gyri and left middle temporal gyrus. IGE patients exhibited prolonged ATT in the left superior temporal gyrus. Furthermore, the prolonged ATT in the left superior temporal gyrus was negatively correlated with the age at onset in IGE patients. These findings suggest that IGE with absence seizures is likely associated with cortical haemodynamic abnormality, providing insight into the pathophysiological mechanism of the complex disease.

Increased CBF was previously reported during the postictal phase of typical absence seizures^27^ and the interictal period of juvenile myoclonic epilepsy^25^. Another study reported that elevated CBF in the amygdala after status epilepticus in rats may continue beyond the sub-acute phase and for up to 14 days, and the increased vessel density observed using immunohistochemistry as the pathophysiological basis of elevated CBF in the amygdala^28^. Postictal structural MRI studies also presented evidence of localized postictal cortical swelling, increased signal intensity on T2-weighted images and blood-brain barrier abnormalities as indicated by abnormal contrast enhancement^29^,^30^,^31^,^32^. ASL hyperperfused levels identified during the postictal phase can suggest underlying damage of the blood–brain barrier due to inflammatory processes in the epileptogenic focus^33^. In fact, an experimental model of epilepsy previously demonstrated that vascular alterations leading to hyperperfusion are inextricably connected to the generation of seizures, possibly mediated by leukocyte-endothelium interaction^33^,^34^. Our results, together with the above evidence, suggest hyperperfused patterns during the postictal phase in IGE patients with absence seizures.

Whether GSW discharges in patients with IGE originates in the cortex, subcortex (e.g., thalamus) or simultaneously in a cortical-subcortical network remains a matter of debate^27^,^35^. In this study, IGE patients exhibited a significantly increased CBF in the left parahippocampal gyrus, bilateral fusiform gyri and left middle temporal gyrus. The parahippocampal gyrus and middle temporal gyrus are both located in the temporal lobe, which is the most common region involved in epilepsy. Cortical morphologic changes in the middle temporal gyrus and fusiform gyrus have been identified in patients with IGE^36^ and juvenile myoclonic epilepsy^37^. Another study on juvenile myoclonic epilepsy revealed that epileptiform discharges have both localized onsets and a restricted cortical network during propagation that includes the regions of temporal and frontal cortex and are not “generalized” in the sense of bilaterally synchronous diffuse onsets^38^. The parahippocampal gyrus, which is anatomically and/or functionally connected with the hippocampus, is currently theorized to be critical for memory storage and long-term memory. Previous studies have demonstrated that IGE patients have memory and cognition deficits^39^,^40^. Although volumetric measurement of the parahippocampal gyrus in patients with IGE did not exhibit significant volume alteration, increased activity in the parahippocampal gyrus was noted on EEG^41^,^42^. This finding is consistent with our result of increased CBF in the parahippocampal gyrus, providing further evidence that memory deficits in IGE may be due to neuronal dysfunction of the parahippocampal gyrus secondary to epileptic activity. Our results suggest that cortical dysfunction in the temporal lobe and fusiform gyrus may be related to epileptic activity in IGE patients with absence seizures.

In addition, the present work used ATT to quantify the variability of cerebral perfusion patterns in IGE patients with absence seizures. ATT is another haemodynamic parameter measured by quantitative ASL with multiple-delay time sampling and represents the duration of the labelled blood flowing from the labelling region to the vascular compartment of the imaging sections^43^. In broad clinical populations, ATT is especially uncertain owing to various pathophysiological alterations of different diseases^16^. In addition, regional variation in CBF and ATT in different brain regions was detected in the normal human and rat brain using continuous arterial spin labelling MRI^16^,^44^. The heterogeneous distribution of ATT in different regions in the normal brain and under normal and pathological conditions is one major confounding issue affecting the accuracy of ASL-based CBF quantification^45^. Clinical use has demonstrated the challenges of optimizing ATT for subjects considering the heterogeneity of ATT acquiring labelled images only at a single post-labelling time point, particularly under pathological conditions^17^,^43^,^46^. In multiple ASL acquisitions at various post-labelling times, all of the labelled spins contribute to the perfusion signal intensity, and no prior knowledge of individual transit times is needed^47^.

Our results revealed that IGE patients with absence seizures showed prolonged ATT in the left superior temporal gyrus. This finding is consistent with a recently published study that found that patients with late-onset epilepsy have significantly prolonged ATT in widespread brain regions, but the greatest prolongation is predominantly distributed in the temporal and frontal lobes^20^. The authors considered that the prolongation of ATT may be attributable to the recruitment of secondary collaterals induced by seizure activity. Another speculative interpretation of ATT prolongation may be caused by cortical inhibition corresponding to decreased synaptic activity after seizure activity, such as that caused by reduced neuronal input. Aghakhani Y *et al*. believed that not all GSW discharges are generated by the same mechanisms and that some brain regions become more active whereas others become less active^1^. In addition, seizure-induced brain abnormalities, such as cortical swelling, T2-weighted hyperintensity and restricted diffusion, have been described as postictal MRI features within 12 hours to 14 days from a seizure^29^,^30^,^31^,^32^,^48^,^49^. These abnormalities following seizure activity may lead to cortical inhibition. Moreover, we detected that prolonged ATT in the left superior temporal gyrus was negatively correlated with the age at onset in IGE patients, suggesting that the pathophysiological alteration was more obvious in the brain region in IGE of earlier onset. Given the complexity of the regulation of cerebral circulation, ATT could provide additional information in addition to CBF to account for cerebral perfusion patterns. Our research acquired the preliminary results of ATT alteration in IGE patients. However, further investigation is needed to confirm these findings and define the pathophysiological mechanisms of prolonged ATT in IGE patients.

There are several limitations to our study that should be acknowledged. First, owing to the small sample size, our results must be confirmed by future studies with a larger sample size. The current study demonstrates the feasibility of using multi-delay multi-parametric ASL perfusion MRI to characterize cerebral perfusion patterns in IGE patients. Second, in our cohort of patients, the different seizure subtypes of IGE are mixed together, including typical absence seizures and absence seizures accompanied by other seizure subtypes of IGE. Thus, these findings should be interpreted with caution because the different seizure subtypes of IGE could lead to inconsistent results. Third, ASL perfusion MRI was performed using a wide time window ranging from 0.5 to 30 days with a mean time of 7.6 days since the last epilepsy episode occurred. Given the complexity of cerebral haemodynamic changes, the cerebral perfusion patterns may vary with different time points after seizures, particularly in the postictal phase. In addition, the through-plane blurring in the single-shot acquisition can affect the 3D-GRASE data and the subsequent perfusion estimates. Finally, simultaneous EEG was not combined with ASL perfusion MRI to analyse the relationship between cerebral haemodynamic changes and GSW discharges in patients with IGE.

Taken together, this study revealed the postictal alterations of regional cerebral perfusion in the brains of IGE patients with absence seizures using multi-delay multi-parametric ASL perfusion MRI with the parameters of ATT and CBF at approximately one week since the last epilepsy episode occurred. Our results demonstrated abnormal perfusion patterns during the postictal phase and provided further evidence that cortical dysfunction in the temporal lobe and fusiform gyrus may be related to epileptic activity in IGE patients with absence seizures. This information can play an important role in elucidating the pathophysiological mechanism of IGE from a cerebral haemodynamic perspective.

## Methods

### Participants

Twenty-one consecutive IGE patients with absence seizures [mean age ± SD: 17.1 ± 4.7 years (range: 9–25 years), 10 males] and 24 age and sex-matched healthy control subjects [mean age ± SD: 19.7 ± 3.5 years (range: 9–24 years); 9 males] were recruited to undergo MRI using a protocol specifically designed for the present study. IGE patients were diagnosed and classified based on clinical examination and EEG according to the International League Against Epilepsy (ILAE) classification^50^.

The inclusion criteria for the patients were as follows: (1) clinically diagnosed absence seizures with or without generalized tonic-clonic seizures and/or juvenile myoclonic epilepsy; (2) experienced more than two spontaneous epileptic seizures; (3) no evidence of secondary generalized seizures, such as tumours, encephalitis, and vascular malformation; (4) no structural or signal abnormalities on conventional brain MRI; (5) normal neurological examination, except for GSW mixtures or generalized spikes with normal alpha rhythms on EEG; (6) patients who had prior antiepileptic treatment received no medication for at least 48 h before the MRI; and (7) right-handedness. The exclusion criteria were as follows: (1) a history of head trauma, psychiatric or other neurological disorders; (2) a history of addictions; (3) a history of partial seizures; (4) self-reported falling asleep during ASL perfusion MRI scanning; (5) excessive head motion exceeding 1.5-mm translation and 1.5° rotation; and (6) any contraindications to MRI scanning. In our cohort, eighteen patients were taking antiepileptic drugs, including valproate, levetiracetam, lamotrigine, oxcarbazepine or some combination. In addition, 3 patients had never taken any medications.

All healthy control subjects were right-handed. No familial or personal histories of neurological or psychiatric diseases were noted. Head trauma or lesions on brain MRI were not noted in healthy control subjects. This study was approved by the West China Hospital Clinical Trials and Biomedical Ethics Committee of Sichuan University, and written informed consent was obtained from all of the participants. The study protocol was performed in accordance with the approved guidelines.

### MRI protocols

The patients and healthy control subjects were imaged using a 3-T Siemens Trio Tim system with a 12-channel phased-array head coil. Multi-delay multi-parametric ASL perfusion MRI was performed using a pCASL pulse sequence with background-suppressed single-shot 3D GRASE readouts. The imaging parameters were as follows: 4 PLD times, 1,400/1,800/2,200/2,600 ms; labelling pulse duration, 1,500 ms; TR, 3,500/3,500/3,500/3,500 ms; TE, 22 ms; voxel size, 3.44 × 3.44 × 5 mm^3^; 26 slices covering the whole brain; 12 pairs of tag/control images were acquired for each delay with a total scan time of 5.9 min. An M0 image was acquired using TR = 5,000 ms and PLD = 4,000 ms (scan time = 15 s). ASL perfusion images were acquired with background suppressed, but M0 image was acquired without background suppression.

### Data processing

SPM8 (Wellcome Trust Centre for NeuroImaging, UCL, UK) and a self-complied Matlab program based on SPM8 (provided by Siemens Healthcare, MR Collaborations NE Asia, Beijing, China) were used for image processing. Motion correction was performed first for all control and label images of the same series using the M0 image as a reference. After motion correction, the mean perfusion difference, ΔM (i), in the images was generated for each PLD. The weighted delay (WD) was calculated using the images of ΔM (i) by equation (1) and converted to ATT based on the theoretical relationship between the WD and ATT as described in Dai W *et al*.^16^ and Wang DJ *et al*.^18^


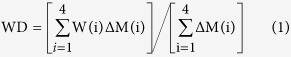


**Figure 3:**
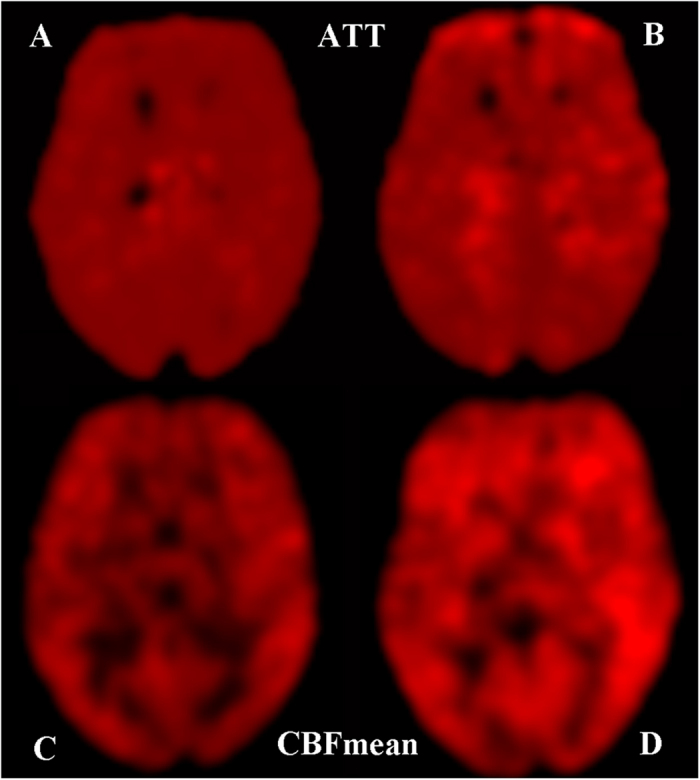
Maps of the ATT (**A,B**) and mean CBF (**C,D**) randomly selected from the participants. The ATT of an IGE patient (**B**) was prolonged in the left temporal lobe in comparison with that in a healthy control subject (**A**). The mean CBF of an IGE patient (**D**) was increased in the left temporal lobe and bilateral frontal lobe in comparison with that in a healthy control subject (**C**).

The CBF at each delay was calculated using the following equation:


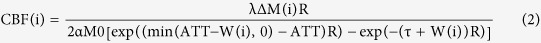


where λ ( = 0.9 g/ml) is the blood/tissue water partition coefficient, R ( = 0.61 s^−1^) is the longitudinal relaxation rate of blood at 3T, α ( = 0.8) is the tagging efficiency, τ ( = 1,500 ms) is the duration of the labelling pulse, and w(i) is the PLD ( = 1,400/1,800/2,200/2,600 ms). The final CBF was the mean of the estimated CBF at each PLD. All of the ASL images of the IGE patients and healthy control subjects were spatially normalized to the Montreal Neurological Institute (MNI) template space with 79 × 95 × 68 dimension and were smoothed using a 6-mm full width at half maximum Gaussian kernel using SPM8. A representative example of the ATT and mean CBF map of an IGE patient and healthy control subject is shown in Fig. 3.

### Statistical analysis

The two-sample t-test was used for the group comparisons of the ATT and CBF differences in the whole brain between the IGE patients and healthy control subjects. The height threshold for the SPM analysis was set to the false discovery rate (FDR) corrected *p* < 0.05 with an extent threshold of 100 voxels to localize those brain regions with changed perfusion. Statistical maps were overlaid onto a high-resolution brain template in the standard MNI space using MRIcron software (http://www.mccauslandcenter.sc.edu/crnl/mricron/index.html). To investigate the relationship between the abnormal cerebral perfusion patterns (i.e., ATT and CBF) and clinical characteristics, the regional values of ATT and CBF in the identified brain areas were extracted using an automated tool (Marsbar, version 0.44; http://marsbar.sourceforge.net). Pearson’s correlation was employed to evaluate the relationships between these neuroimaging findings and illness duration, as well as the age at onset (*p* < 0.05). The statistical analyses were performed using SPSS 20.0 software (SPSS Statistics, IBM, Armonk, NY).

## Additional Information

**How to cite this article**: Chen, G. *et al*. Patterns of postictal cerebral perfusion in idiopathic generalized epilepsy: a multi-delay multi-parametric arterial spin labelling perfusion MRI study. *Sci. Rep.* **6**, 28867; doi: 10.1038/srep28867 (2016).

## Figures and Tables

**Table 1:** Characteristics of the participants.

	IGE with absence seizures (n = 21)	Healthy control subjects (n = 24)	*p*-value
Gender (male/female)^a^	10/11	9/15	0.493
Age (years)^b^	17.1 ± 4.7	19.7 ± 3.5	0.063
Age at onset (years)	12.0 ± 3.8		
Illness duration (years)	5.2 ± 4.5		
Postictal duration of last seizure activity before MRI (days)	7.6 ± 5.9		

IGE: idiopathic generalized epilepsy.

^a^Chi- squared test was used for gender comparisons between the IGE patients and healthy control subjects.

^b^Two sample two-tailed t-test was used for age comparisons between the IGE patients and healthy control subjects.

**Table 2:** Regional cerebral perfusion difference demonstrated by multi-delay multi-parametric ASL perfusion MRI between IGE patients with absence seizures and healthy control subjects.

	Region	Side	MNI coordinates			Cluster	*p*-value^a^
			x	y	z		
ATT	Superior temporal gyrus	L	−62	−52	18	372	0.038
CBF_mean_	Middle temporal gyrus	L	−58	−68	8	658	0.007
	Parahippocampal gyrus	L	−24	−34	−16	285	0.019
	Fusiform gyrus	L	−32	−56	−8	199	0.022
CBF_1,400_	Parahippocampal gyrus Fusiform gyrus	L	−30	−54	−8	411	0.022
	Fusiform gyrus	R	36	−52	−12	262	0.022
	Middle temporal gyrus	L	−66	−50	−8	173	0.022
CBF_1,800_	Middle temporal gyrus	L	−62	−60	6	123	0.036

ASL: arterial spin labelling; ATT: arterial transit time; CBF: cerebral blood flow; MRI: magnetic resonance imaging; IGE: idiopathic generalized epilepsy; MNI: Montreal neurological institute; L: left; R: right.

^a^*p*-values were corrected for the false discovery rate (*p* < 0.05).

**Table 3:** Correlation between the abnormal cerebral perfusion patterns (ATT and CBF) and illness duration, as well as the age at onset in IGE patients with absence seizures.

	Region	Side	Illness duration		Age at onset	
			*r*	*p*-value	*r*	*p*-value
ATT	Superior temporal gyrus	L	0.019	0.933	−0.504	0.020*
CBF_mean_	Middle temporal gyrus	L	0.421	0.058	−0.427	0.054
	Parahippocampal gyrus	L	0.205	0.373	0.004	0.986
	Fusiform gyrus	L	0.272	0.233	−0.240	0.295
CBF_1,400_	Parahippocampal gyrus Fusiform gyrus	L	0.077	0.742	−0.012	0.959
	Fusiform gyrus	R	0.015	0.950	0.099	0.669
	Middle temporal gyrus	L	0.202	0.380	−0.372	0.097
CBF_1,800_	Middle temporal gyrus	L	0.346	0.125	−0.367	0.102

ATT: arterial transit time; CBF: cerebral blood flow; IGE: idiopathic generalized epilepsy; L: left; R: right; *p < 0.05.

## References

[b1] Aghakhani Y. . fMRI activation during spike and wave discharges in idiopathic generalized epilepsy. Brain 127, 1127–1144 (2004). 15033899 10.1093/brain/awh136

[b2] Duncan J. S. Brain imaging in idiopathic generalized epilepsies. Epilepsia 46 Suppl 9, 108–111 (2005). 16302883 10.1111/j.1528-1167.2005.00321.x

[b3] Williams D. S., Detre J. A., Leigh J. S. & Koretsky A. P. Magnetic resonance imaging of perfusion using spin inversion of arterial water. Proc Natl Acad Sci USA 89, 212–216 (1992). 1729691 10.1073/pnas.89.1.212 PMC48206

[b4] Hales P. W., Kawadler J. M., Aylett S. E., Kirkham F. J. & Clark C. A. Arterial spin labeling characterization of cerebral perfusion during normal maturation from late childhood into adulthood: normal ‘reference range’ values and their use in clinical studies. J Cereb Blood Flow Metab 34, 776–784 (2014). 24496173 10.1038/jcbfm.2014.17 PMC4013758

[b5] Preibisch C. . Age-related cerebral perfusion changes in the parietal and temporal lobes measured by pulsed arterial spin labeling. J Magn Reson Imaging 34, 1295–1302 (2011). 21953683 10.1002/jmri.22788

[b6] Lui S. . Depressive disorders: focally altered cerebral perfusion measured with arterial spin-labeling MR imaging. Radiology 251, 476–484 (2009). 19401575 10.1148/radiol.2512081548

[b7] Oishi M. . Ictal focal hyperperfusion demonstrated by arterial spin-labeling perfusion MRI in partial epilepsy status. Neuroradiology 54, 653–656 (2012). 22418862 10.1007/s00234-012-1027-7

[b8] Pendse N. . Interictal arterial spin-labeling MRI perfusion in intractable epilepsy. J Neuroradiol 37, 60–63 (2010). 19674791 10.1016/j.neurad.2009.05.006

[b9] Matsuura K. . Usefulness of arterial spin-labeling images in periictal state diagnosis of epilepsy. J Neurol Sci 359, 424–429 (2015). 26478131 10.1016/j.jns.2015.10.009

[b10] Guo X. . Asymmetry of cerebral blood flow measured with three-dimensional pseudocontinuous arterial spin-labeling MR imaging in temporal lobe epilepsy with and without mesial temporal sclerosis. J Magn Reson Imaging 42, 1386–1397 (2015). 25884243 10.1002/jmri.24920

[b11] Wolf R. L. . Detection of mesial temporal lobe hypoperfusion in patients with temporal lobe epilepsy by use of arterial spin labeled perfusion MR imaging. AJNR Am J Neuroradiol 22, 1334–1341 (2001). 11498422 PMC7975208

[b12] Watts J. M., Whitlow C. T. & Maldjian J. A. Clinical applications of arterial spin labeling. NMR Biomed 26, 892–900 (2013). 23378178 10.1002/nbm.2904

[b13] Telischak N. A., Detre J. A. & Zaharchuk G. Arterial spin labeling MRI: clinical applications in the brain. J Magn Reson Imaging 41, 1165–1180 (2015). 25236477 10.1002/jmri.24751

[b14] Sierra-Marcos A. . Accuracy of arterial spin labeling magnetic resonance imaging (MRI) perfusion in detecting the epileptogenic zone in patients with drug-resistant neocortical epilepsy: comparison with electrophysiological data, structural MRI, SISCOM and FDG-PET. Eur J Neurol 23, 160–167 (2016). 26346555 10.1111/ene.12826

[b15] Wang R. . Multi-delay arterial spin labeling perfusion MRI in moyamoya disease--comparison with CT perfusion imaging. Eur Radiol 24, 1135–1144 (2014). 24557051 10.1007/s00330-014-3098-9 PMC4143230

[b16] Dai W., Robson P. M., Shankaranarayanan A. & Alsop D. C. Reduced resolution transit delay prescan for quantitative continuous arterial spin labeling perfusion imaging. Magn Reson Med 67, 1252–1265 (2012). 22084006 10.1002/mrm.23103 PMC3367437

[b17] Johnston M., Lu K., Maldjian J. & Jung Y. Multi-TI arterial spin labeling MRI with variable TR and bolus duration for cerebral blood flow and arterial transit time mapping. IEEE Trans Med Imaging 34, 1392–1402 (2015). 25616010 10.1109/TMI.2015.2395257

[b18] Wang D. J. . Multi-delay multi-parametric arterial spin-labeled perfusion MRI in acute ischemic stroke - Comparison with dynamic susceptibility contrast enhanced perfusion imaging. Neuroimage Clin 3, 1–7 (2013). 24159561 10.1016/j.nicl.2013.06.017 PMC3791289

[b19] Martin S. Z. . 3D GRASE pulsed arterial spin labeling at multiple inflow times in patients with long arterial transit times: comparison with dynamic susceptibility-weighted contrast-enhanced MRI at 3 Tesla. J Cereb Blood Flow Metab 35, 392–401 (2015). 25407272 10.1038/jcbfm.2014.200 PMC4348376

[b20] Hanby M. F. . Structural and physiological MRI correlates of occult cerebrovascular disease in late-onset epilepsy. Neuroimage Clin 9, 128–133 (2015). 26413475 10.1016/j.nicl.2015.07.016 PMC4556750

[b21] Prevett M. C., Duncan J. S., Jones T., Fish D. R. & Brooks D. J. Demonstration of thalamic activation during typical absence seizures using H_2_^15^O and PET. Neurology 45, 1396–1402 (1995). 7617203 10.1212/wnl.45.7.1396

[b22] Joo E. Y., Tae W. S. & Hong S. B. Cerebral blood flow abnormality in patients with idiopathic generalized epilepsy. J Neurol 255, 520–525 (2008). 18283401 10.1007/s00415-008-0727-8

[b23] Kapucu L. O., Serdaroglu A., Okuyaz C., Kose G. & Gucuyener K. Brain single photon emission computed tomographic evaluation of patients with childhood absence epilepsy. J Child Neurol 18, 542–548 (2003). 13677580 10.1177/08830738030180080401

[b24] Yeni S. N., Kabasakal L., Yalcinkaya C., Nisli C. & Dervent A. Ictal and interictal SPECT findings in childhood absence epilepsy. Seizure 9, 265–269 (2000). 10880286 10.1053/seiz.2000.0400

[b25] Tae W. S., Joo E. Y., Han S. J., Lee K. H. & Hong S. B. CBF changes in drug naive juvenile myoclonic epilepsy patients. J Neurol 254, 1073–1080 (2007). 17351720 10.1007/s00415-006-0491-6

[b26] Bek S. . Lateralization of cerebral blood flow in juvenile absence seizures. J Neurol 257, 1181–1187 (2010). 20155275 10.1007/s00415-010-5488-5

[b27] Nehlig A. . Ictal and interictal perfusion variations measured by SISCOM analysis in typical childhood absence seizures. Epileptic Disord 6, 247–253 (2004). 15634621

[b28] Hayward N. M., Ndode-Ekane X. E., Kutchiashvili N., Grohn O. & Pitkanen A. Elevated cerebral blood flow and vascular density in the amygdala after status epilepticus in rats. Neurosci Lett 484, 39–42 (2010). 20709151 10.1016/j.neulet.2010.08.013

[b29] Silverstein A. M. & Alexander J. A. Acute postictal cerebral imaging. AJNR Am J Neuroradiol 19, 1485–1488 (1998). 9763382 PMC8338683

[b30] Hicdonmez T., Utku U., Turgut N., Cobanoglu S. & Birgili B. Reversible postictal MRI change mimicking structural lesion. Clin Neurol Neurosurg 105, 288–290 (2003). 12954549 10.1016/s0303-8467(03)00047-7

[b31] Goyal M. K., Sinha S., Ravishankar S. & Shivshankar J. J. Peri-ictal signal changes in seven patients with status epilepticus: interesting MRI observations. Neuroradiology 51, 151–161 (2009). 19057899 10.1007/s00234-008-0479-2

[b32] Fabene P. F., Marzola P., Sbarbati A. & Bentivoglio M. Magnetic resonance imaging of changes elicited by status epilepticus in the rat brain: diffusion-weighted and T2-weighted images, regional blood volume maps, and direct correlation with tissue and cell damage. Neuroimage 18, 375–389 (2003). 12595191 10.1016/s1053-8119(02)00025-3

[b33] Storti S. F. . Combining ESI, ASL and PET for quantitative assessment of drug-resistant focal epilepsy. Neuroimage 102, 49–59 (2014). 23792219 10.1016/j.neuroimage.2013.06.028

[b34] Fabene P. F. . A role for leukocyte-endothelial adhesion mechanisms in epilepsy. Nat Med 14, 1377–1383 (2008). 19029985 10.1038/nm.1878 PMC2710311

[b35] McGill M. L. . Functional neuroimaging abnormalities in idiopathic generalized epilepsy. Neuroimage Clin 6, 455–462 (2014). 25383319 10.1016/j.nicl.2014.10.008 PMC4221627

[b36] Betting L. E. . Correlation between quantitative EEG and MRI in idiopathic generalized epilepsy. Hum Brain Mapp 31, 1327–1338 (2010). 20082332 10.1002/hbm.20944 PMC6870673

[b37] Ronan L. . Widespread cortical morphologic changes in juvenile myoclonic epilepsy: evidence from structural MRI. Epilepsia 53, 651–658 (2012). 22360759 10.1111/j.1528-1167.2012.03413.x

[b38] Holmes M. D., Quiring J. & Tucker D. M. Evidence that juvenile myoclonic epilepsy is a disorder of frontotemporal corticothalamic networks. Neuroimage 49, 80–93 (2010). 19679190 10.1016/j.neuroimage.2009.08.004

[b39] Dickson J. M., Wilkinson I. D., Howell S. J., Griffiths P. D. & Grunewald R. A. Idiopathic generalised epilepsy: a pilot study of memory and neuronal dysfunction in the temporal lobes, assessed by magnetic resonance spectroscopy. J Neurol Neurosurg Psychiatry 77, 834–840 (2006). 16574726 10.1136/jnnp.2005.086918 PMC2117498

[b40] Realmuto S. . Social cognition dysfunctions in patients with epilepsy: Evidence from patients with temporal lobe and idiopathic generalized epilepsies. Epilepsy Behav 47, 98–103 (2015). 25982884 10.1016/j.yebeh.2015.04.048

[b41] Zhou S. Y. . Selective medial temporal volume reduction in the hippocampus of patients with idiopathic generalized tonic-clonic seizures. Epilepsy Res 110, 39–48 (2015). 25616454 10.1016/j.eplepsyres.2014.11.014

[b42] Clemens B. . EEG-LORETA endophenotypes of the common idiopathic generalized epilepsy syndromes. Epilepsy Res 99, 281–292 (2012). 22240326 10.1016/j.eplepsyres.2011.12.008

[b43] Yoshiura T. . Simultaneous measurement of arterial transit time, arterial blood volume, and cerebral blood flow using arterial spin-labeling in patients with Alzheimer disease. AJNR Am J Neuroradiol 30, 1388–1393 (2009). 19342545 10.3174/ajnr.A1562 PMC7051557

[b44] Thomas D. L., Lythgoe M. F., van der Weerd L., Ordidge R. J. & Gadian D. G. Regional variation of cerebral blood flow and arterial transit time in the normal and hypoperfused rat brain measured using continuous arterial spin labeling MRI. J Cereb Blood Flow Metab 26, 274–282 (2006). 16034369 10.1038/sj.jcbfm.9600185 PMC2702127

[b45] Qin Q. . Three-dimensional whole-brain perfusion quantification using pseudo-continuous arterial spin labeling MRI at multiple post-labeling delays: accounting for both arterial transit time and impulse response function. NMR Biomed 27, 116–128 (2014). 24307572 10.1002/nbm.3040 PMC3947417

[b46] MacIntosh B. J. . Multiple inflow pulsed arterial spin-labeling reveals delays in the arterial arrival time in minor stroke and transient ischemic attack. AJNR Am J Neuroradiol 31, 1892–1894 (2010). 20110375 10.3174/ajnr.A2008 PMC7964001

[b47] Bokkers R. P. . Arterial spin-labeling MR imaging measurements of timing parameters in patients with a carotid artery occlusion. AJNR Am J Neuroradiol 29, 1698–1703 (2008). 18701581 10.3174/ajnr.A1232 PMC8118771

[b48] Cianfoni A. . Seizure-induced brain lesions: a wide spectrum of variably reversible MRI abnormalities. Eur J Radiol 82, 1964–1972 (2013). 23787273 10.1016/j.ejrad.2013.05.020

[b49] Xiang T., Li G., Liang Y. & Zhou J. A wide spectrum of variably periictal MRI abnormalities induced by a single or a cluster of seizures. J Neurol Sci 343, 167–172 (2014). 24950900 10.1016/j.jns.2014.06.001

[b50] Engel J. Jr. A proposed diagnostic scheme for people with epileptic seizures and with epilepsy: report of the ILAE task force on classification and terminology. Epilepsia 42, 796–803 (2001). 11422340 10.1046/j.1528-1157.2001.10401.x

